# A Physiological Conception of Prolonged Hysterical Conditions
1Paper read at meeting of the Bristol Medico-Chirurgical Society, on December 14th, 1921.


**Published:** 1922-06

**Authors:** H. H. Carleton


### A PHYSIOLOGICAL CONCEPTION OF PROLONGED HYSTERICAL CONDITIONS.[1]

BY

H. H. Carleton, M.A., M.D. Oxon.

In any discussion on hysteria some psychological treatment cannot be avoided, but it is upon the physiological aspects of the subject that I wish to lay stress since the physiological disturbances are most pronounced when hysterical states have been in existence for long periods.

In recent years most of us became familiar with transient hysterical symptoms, which, under appropriate treatment, were quickly cured. Observations upon large numbers of soldiers have thrown much light upon the nature of hysteria and various definitions of hysteria have been evolved. One may cite Hurst's definition as fairly representing one section of medical opinion some three years ago. Hysteria, Hurst

[1] Paper read at meeting of the Bristol Medico-Chirurgical Society, on December 14th, 1921.

said, is a condition in which symptoms are present, which are produced by suggestion and which are curable by persuasion or psychotherapy; the essence of such a definition being that hysteria consists of the symptoms and nothing more; remove these, and you have a normal individual.

This modern conception of hysteria was in contradistinction to the older view of Charcot, who regarded hysteria as an enduring state, which could be detected, if looked for, by the presence of so-called stigmata; and that the subject of this chronic state revealed himself or herself in true colours from time to time by the development of exacerbations, namely acute hysterical symptoms. Babinski and others recognised that stigmata were for the most part created by the examining physician, and as a result Charcot's view fell into disrepute.

With the return swing of the pendulum, however, tribute was paid to Charcot's conception by certain schools of psychological medicine, which recognised the mental background of the hysteric as an important and lasting factor. These observers realised that the removal of the symptom did not necessarily or even usually cure the patient.

Clinical evidence all goes to favour the view that in many cases of hysteria physiological changes of an enduring nature are liable to occur and exhibit their presence both in the neuromuscular and the visceral and vascular systems of patients. These changes render the hysteric physically, as well as mentally, different from the normal individual and often difficult to cure.

The clinical complex hysteria includes, then, both a mental state and physical symptoms. The latter, since they afford obvious objective evidence, generally arrest attention. It is the relationship between the mental process, the overflow of nervous energy into the sympathetic nervous system, the resultant endocrine disturbance, and the recoil, so to speak, of this upon the central nervous system, that comprise the essential factors in prolonged hysterical conditions.

The following case, which is a somewhat extreme type, may be taken as an example. Milder cases differ in degree only, and can be brought into line with such a general conception as I hope to lay before you.

A case of severe hysteria has been recently under my care, a man aged 30 years, wildly ataxic and spastic in legs, arms and body. He was so profoundly apprehensive that he started excessively at the slightest unexpected sound. The gross motor disturbances were accompanied by marked changes in the field of autonomic nervous control, that is in the visceral and vascular systems and in the endocrine glands. Manifestations of such changes included vasomotor instability, excessive sweating, tachycardia, exophthalmos, rapid breathing, anorexia, constipation, etc.

These changes require consideration from the point of view of certain physiological conceptions of an integrated nervous system.

First of all, let us turn our attention to the motor symptoms, ataxy and spasm. On examination, unequivocal signs of organic nerve disease were entirely wanting.

What, then, was the essential basis of this loss of equilibrium and lack of co-ordination ?

Normally, when we are standing still or when we walk our static or kinetic equilibrium is assured by a series of tonic muscular contractions, which are integrated and adapted in segmental spinal, cerebellar and labyrinthine reflex arcs, named by Sherrington the proprioceptive system. This cerebellar system is adjuvant to, and subject to the control of, the highest levels of the nervous hierarchy, the cerebral system.

Nervous integration is so delicately adapted and complete, that in a given situation the tonic contractions are automatically increased or diminished. Once learnt, equilibration recedes from attentive levels of consciousness and is harmoniously merged into a wider scheme of behaviour. The attentive level, at any particular moment, is the most comprehensive environmental field to which the organism is responding. All other aspects of the environment (to which subservient behaviour systems are responding at the time) are subconscious. They are in consciousness, but not in the upper level of attention. For instance, if I stop thinking about a business engagement to which my legs are carrying me I can *consciously* walk, if I cease to do this I can *consciously* take a step, ceasing this I can *consciously* merely equilibrate in the upright posture, or ceasing this I can become *conscious* of pressure on the soles of my feet. The stream of consciousness is simply this selected procession of environmental aspects, to which the body's ever varying motor adjustments are directed.

What bearing has all this upon the condition of the hysteric ?

Well, as a result of shock, anxiety, or emotion the hysteric is responding to a very restricted environmental field. Adjustments which in health are unconscious or automatic now occupy the highest level of attention. For instance, hysterics, probably owing to their vasomotor instability, are very prone to dizziness. This dizziness occupies the highest available levels of attention, and leads to false integration of sensory impressions. For the hysteric the ground moves and sways. No longer is walking automatic and controlled at unconscious levels ; it has become a painfully conscious experience. A single step, equilibration even, has become an adventure. The hysteric who tries to walk, because he is convinced of the necessity to make the experiment, at first dares not to put one foot in front of the other. Then he plunges forward, and all at once takes such a stride that he loses his balance. It is practically the same as stepping off into the air, an act which will throw the best balanced person to the ground. More timid than ever, he tries to widen his base of support and straddles his legs in such a way that they resemble the lines of an arrow head. Then he tries to make a forward movement. It goes without saying that the very position he has taken up disturbs his centre of gravity, so that he cannot perform further movement without loss of balance.

Other patients begin by stiffening up all their muscles ; my patient showed this second type of inco-ordination in an extreme degree. He would momentarily relax on one side in order to advance. His efforts were rendered futile by the simultaneous contraction of the antagonists on the other side. In this type of neuropath, therefore, we are not dealing with a true ataxia of central or peripheral origin, but with motor inco-ordinations, based on false integration of sensory impressions, and the common characteristic of this kind of motor disturbance is over-shooting the mark.

Having briefly reviewed the condition as a disorder of behaviour, let us turn and see if we can discover any of the physiological correlates of the disturbance.

Apparently among other things the whole of the proprioceptive system, which deals with the automatic adjustment of deep muscle sense and integrates it with labyrinthine impressions, is entirely out of gear, and the cerebrum is making a poor effort to carry on in the absence of its chief of staff, the cerebellum.

The nervous system is built up of neurones arranged or integrated into patterns, which by their specific activity secure efficient response to environmental change. Inherited from countless generations of ancestors are found certain firmly-established neurone patterns, which we call instincts

With increased personal experience, that is to say, with the gradually developing capacity of the individual to respond to an increasingly complex environment, these original inherited dispositions, or neurone patterns, are subjected to modification, owing to conflict which arises between them and other more recently acquired patterns. These conflicts and modifications of developed and developing neurone patterns constitute the physiological basis of inhibitions. Now inhibitions are the very foundation of those disturbances in the sympathetic nervous system which manifest themselves as affective tones, pleasure or pain.

If one could conceive an animal with its nervous system so simply constituted that in it no neurone pattern ever conflicted with another, there would be no inhibitions. A stimulus would give rise to an appropriate motor response unaccompanied by any disagreable affective tone or feeling. Where flight into safety is easy the presence of danger gives rise to no fear ; but if flight is obstructed all gradations of affective tone from fear to terror result. If a soldier under fire can retreat to his dug-out he may appear entirely unconcerned by the shelling, whereas the same man caught in the open in an air-raid will experience " the wind up " to a high degree.

Without labouring the point, I think we may take it that it is the inhibition of neurone patterns *inter se* which is responsible for the affective or emotional aspects of experience.

Mental conflict and the development of excess of emotion is the curse of the hysteric. This leads to enduring physical stress and the physiological disturbances which are now under discussion.

What is the relationship between emotion and physiological processes ?

When affect or feeling is aroused there is an overflow of nervous energy into the sympathetic nervous system. All of us are familiar with sudden vascular and visceral responses in the field of the autonomic nervous system when our feelings are powerfully aroused. Flushing or pallor, palpitation, increased respiration, sweating, etc., constitute not infrequently an almost instantaneous response to some exciting experience.

It is necessary to remember in this connection that the sympathetic or autonomic nervous system controls also those important structures the endocrine glands. The work of Cannon has established on a sure footing the relationship between anger and fear, etc., and the activity of the suprarenal glands. The manifestations of hyper- and hypothyroid metabolism in relation to the emotional disturbance are too familiar to need more than mention. Cushing and others have enlarged our knowledge of the relationship between the pituitary and other ductless glands and certain metabolic disturbances, characterised, among other phenomena, by mental changes.

In profound emotional states over-activity of certain endocrine glands is frequently very lasting, and is apparently responsible for the enduring nature of the neuro-muscular disturbances so often seen in hysterics and so difficult to cure.

Under conditions of great emotional stress endocrine activity is entirely physiological ; it is essential to successful reaction, essential even to life. This is seen in the turmoil of battles and in the excitement of athletic contests. A good pair of suprarenals may help to win a V.C. ; the rowing man stays the course by reason of his second wind, but the suprarenals supply the second wind. So much for the endocrine as a good servant. But the good servant may make a very bad master. Such does the endocrine gland become when it dominates the organism with its excessive activity.

The patient who is the subject of prolonged hysterical disturbance is thus dominated by his endocrines, notably by the thyroid and suprarenals. This endocrine disturbance is an important accompaniment of such motor symptoms as tremor, ataxy and spasm. These motor symptoms induce or increase apprehension and anxiety, which emotional changes in their turn lead to further excess of endocrine secretion. Thus a vicious circle is established.

Now both psychically and physiologically the hysteric's system is in a state of dissociation. If dissociation means anything on the physiological side, surely it means that there is a disturbance of synaptic relations, an increased resistance at synaptic junctions. The seat of action of certain substances, such as strychnine and tetanus toxin, is the synapse. It seems also as though excess of endocrine substance is powerful to increase resistance at the synapse, so that a functional dissociation occurs.

I go further and suggest that the delicate and complex synaptic junctions, where paths, efferent from the cerebellum, by the superior peduncles through the crossed rubrothalamic region to the cerebrum and mesencephalon, are particularly prone to suffer from this physiological dissociation or blocking.

Why do I say this ? The cerebellar system, we must remember, is built up on the afferent side of the central nervous system, a great appendage so to speak, with specific functions, sharing responsibilities, but with no direct efferent spinal tract of its own. It has no efferent outlet independent of the great projection tracts of the motor cortex and mid-brain.

May I be allowed to use an analogy to illustrate roughly what I conceive to be the general relationship of the cerebeliar system to the cerebrum and remainder of the central nervous system.

Consider for a moment a man on horseback. The rider in the saddle I compare to the cerebellum. He is very directly *en rapport* with his environment, that is to say his afferent relations are ample, but his powers of response are less direct, he must transmit his reactions through the reins to the horse, which ultimately decides the exact nature of the response. Drop the reins or dislodge the rider and co-ordination breaks down.

To return to our hysteric: dissociation which removes cerebellar influence leaves the cerebral levels overloaded with details of adjustment. The patient cannot attend to all these details simultaneously. He narrows down his field of attention and concentrates all his effort upon glaring defects of adjustment that trouble him most. He soon gives up the unequal task and becomes paraplegic, or develops a fantastic gait in his endeavour to circumvent some difficulty of his own creation.

A bad administrator is one who cannot or does not trust his juniors. He becomes inefficient and a muddler because he tries to attend to every small detail of business himself. He no longer possesses a comprehensive field, but his mind contains small restricted patches, isolated and incoherent.

Such would be the condition of the hysteric, but that he usually selects one particular item in experience for his undivided attention. How would the Navy have got on at Jutland if Admiral Beatty had sat in his cabin assiduously polishing his buttons?

This narrowing of attention is often strikingly obvious during the process of re-education of the hysteric, and is responsible for profound anxiety, inefficiency and consequent emotional disturbances, which result in further endocrine over-activity.

I have confined my remarks thus far to a type of case in which the great proprioceptive system is out of gear. A little consideration will show how disturbances in the exteroceptive field may lead to comparable dissociation of synaptic relations. The organs belonging to the exteroceptive field are eyes, ears, nose and skin ; all of us are familiar with hysterical blindness, deafness and anæsthesia.

Here motor disturbances are inconspicuous, and therefore the anxiety condition is of a milder degree. Cures are proportionately easier to effect and relapses are more rare.

If enduring anxiety and endocrine disturbance is the basis of prolonged hysterical conditions, how are we to take them into our reckoning when attempting to effect a cure ? Let me refer for a moment to a useful conception of "suggestion" and "counter-suggestion." It follows from what I have said that unfavourable or harmful suggestion is the imposition of an idea which narrows the patient's field of conscious reaction, until systems which should be subservient in any comprehensive scheme of behaviour dominate consciousness. Favourable counter-suggestion, on the other hand, is the imposition of an idea which expands this field of attention and renders it adequately comprehensive.

Anxiety lest he fall may make the ataxic patient paraplegic ; don't let him flounder about, therefore, on the theory that the hysteric won't hurt himself, because he not infrequently does ; but worse than that, you will forfeit all confidence that he may have placed in you. Guard him carefully in the early stages, and insist that the grossest faults of posture and action be corrected, but don't carry your criticisms of his faults of gait to excess, for by so doing you are narrowing down his field of attention too much. The golfer who is told to alter his stance may think of this and forget to swing properly, then reminded of the latter fault he begins to press, and so his faults accumulate *ad infinitum*. But let him have a ball on the match and a worthy opponent, then his stance, swing and tendency to press will take care of themselves.

So in the re-education of hysterical ataxy the stage quickly comes when the patient's field of attention must be expanded to respond to a comprehensive environmental field. The last stage of cure may be to make the patient run at top speed. The supreme demand, to run, entails enlargement of the field of response to such a degree that there is no time left for the patient to trouble about small details of gait, and normal progression results.

We are all familiar with some of the difficulties of the stammerer. When at ease he stammers little, the fear of stammering makes him inarticulate. Many a stammerer put through voice production exercises becomes hopeless; enlarge his field of attention and teach him to ignore his stammer, and he gets on quite well with his talking.

In conclusion, let me refer to a class of case which is frequently the subject of divided opinion on medical boards. Not infrequently it is affirmed that such and such a tremulous, paraplegic man is a malingerer, because he has frequently been seen walking normally when he did not know he was under observation. In justice to such patients we must remember that they are the victims of easily aroused anxiety and emotion, and that a relapse is their normal response to emotion, without any bad faith on their part, and that the relapse may reasonably be accounted for in terms of endocrine activity and physiological dissociation such as I have endeavoured to describe. The Medical Officer sitting in judgment behind the table at a medical board may be a powerful suggestive agent for evil.

